# The Study of Interactions between Active Compounds of Coffee and Willow (*Salix* sp.) Bark Water Extract

**DOI:** 10.1155/2014/386953

**Published:** 2014-06-11

**Authors:** Agata Durak, Urszula Gawlik-Dziki

**Affiliations:** Department of Biochemistry and Food Chemistry, University of Life Sciences, Skromna Street 8, 20-704 Lublin, Poland

## Abstract

Coffee and willow are known as valuable sources of biologically active phytochemicals such as chlorogenic acid, caffeine, and salicin. The aim of the study was to determine the interactions between the active compounds contained in water extracts from coffee and bark of willow (*Salix purpurea* and *Salix myrsinifolia*). Raw materials and their mixtures were characterized by multidirectional antioxidant activities; however, bioactive constituents interacted with each other. Synergism was observed for ability of inhibition of lipid peroxidation and reducing power, whereas compounds able to scavenge ABTS radical cation acted antagonistically. Additionally, phytochemicals from willow bark possessed hydrophilic character and thermostability which justifies their potential use as an ingredient in coffee beverages. Proposed mixtures may be used in the prophylaxis or treatment of some civilization diseases linked with oxidative stress. Most importantly, strong synergism observed for phytochemicals able to prevent lipids against oxidation may suggest protective effect for cell membrane phospholipids. Obtained results indicate that extracts from bark tested *Salix* genotypes as an ingredient in coffee beverages can provide health promoting benefits to the consumers; however, this issue requires further study.

## 1. Introduction

Multitarget therapy is a new therapy concept which tries to treat diseases with a multidrug combination in a more causally directed manner. Physicians practicing phytotherapy recognized very early that a greater efficacy can be achieved with the application of a combination of plant extracts than with a (usually high dosed) monodrug. They noticed that this therapy concept at the same time has the advantage of reducing or eliminating side effects due to the lower doses of the single compounds or drug components within the extract mixtures [1, 2].

Results of synergy effects have been described using two mathematical equations. According to the first equation: *E*(*d*_*a*_, *d*_*b*_) > *E*(*d*_*a*_) + *E*(*d*_*b*_), “a total effect of a combination is greater than expected from the sum of the effects of the single components.” The second equation states that “synergy is deemed present if the effect of a combination is greater than that of each of the individual agents” (i.e., *E*(*d*_*a*_, *d*_*b*_) > *E*(*d*_*a*_) and *E*(*d*_*a*_, *d*_*b*_) > *E*(*d*_*b*_); *E* = observed effect and *d*_*a*_ and *d*_*b*_ are the doses of agents *a* and *b*) [3].

Coffee is a popular beverage that is widely consumed around the world [4, 5]. Recently, scientific studies have pointed out the positive effect of coffee on human health [6]. However, there are some reports with little evidence of health risks and considerable evidence of health benefits for healthy adults as a result of moderate coffee consumption [7]. The beverage also stands out as a dietary source of potential antioxidants, such as caffeine [8, 9], chlorogenic acids [10, 11], hydroxycinnamic acids [12], and Maillard reaction products, such as melanoidins [13, 14]. Thus, the antioxidant capacity of coffee is related to the presence of both natural constituents and compounds formed during processing. Most studies in the literature refer to the antioxidant activity of roasted coffee. Despite the great economic importance of soluble coffee, little has been reported about its antioxidant potential or the influence of processing conditions. To become solubilised, coffee undergoes an extraction process. The beans are roasted, ground, and subjected to successive percolation with water at temperatures ranging from 100 to 180°C. Chemically, percolation results in the selective solubilisation of coffee solids. Depolymerisation and degradation of coffee solids may occur during high-temperature extraction [15], and process variations may affect the product's characteristics.

Willow bark is included in the Polish Pharmacopeia and in the European monograph as a constituent of many herbal drugs, dietary supplements, and weight loss enhancement remedies [16–18]. Willow bark preparations, better tolerated by patients than synthetic derivatives, can be used for the traditional symptomatic indications of fever, infections, mild rheumatic complaints, headache, and chronic pain syndromes and extracts of bark have antioxidant abilities, thus many people have begun to turn back to willow bark as an alternative to aspirin [18–22].

*Salix purpurea* L.,* S. daphnoides* Vill., and* S. alba* L. are a very popular herbal species affirmed in the natural habitats and field-cultivated in Poland [23, 24]. Bark of this species contains phenolic glicosides, **ρ**-hydroxybenzoic, vanillic, cinnamic,  **ρ**-coumaric, ferulic, and caffeic acids and naringenin known for their prohealth properties [19, 25, 26]. However, little is as yet known about synergic effects of coffee and willow bark bioactive compounds.

It has been hypothesized that interactions between the active compounds may improve the health promoting properties of both materials. Thus, the aim of the study was to determine the interaction between antioxidant compounds contained in soluble coffee and present in the extracts from the bark of some species of willow (*Salix purpurea* and* Salix myrsinifolia*).

## 2. Materials and Methods 

### 2.1. Chemicals

ABTS (2,2′-azino-bis(3-ethylbenzothiazoline-6-sulphonic acid)), Folin-Ciocalteu reagent, gallic acid, linoleic acid, and potassium ferricyanide were purchased from Sigma-Aldrich company (Poznan, Poland). Acetonitrile and methanol gradient HPLC grade and formic acid LC-MS grade for LC-UV-MS separations were purchased from J. T. Baker (Phillipsburg, New Jersey, USA). Water was purified in-house with a Milli-Q water purification system Simplicity-185 (EMD Millipore Corporation, Billerica, Massachusetts, USA). All other chemicals were of analytical grade.

### 2.2. Materials

*Salix purpurea* and* Salix myrsinifolia *plants were cultivated on the sandy soil (heavy loamy sand) of experimental fields at the University of Life Sciences in Lublin (51°33′N; 22°44′E). Willow shoots were harvested in November (2012) in three replicates. The shoots were washed with deionized water. Bark was separated from the wood by peeling and subsampled for chemical analysis. The bark material sampled for salicylate analysis was dried at room temperature and intensively mixed and homogenized. After drying, the phenolic glycosides content calculated on salicin was determined by means of the HPLC technique in the laboratory of Labofarm in Starogard Gdański according to methods in Polish Pharmacopoeia VI (2002) and expressed as mg/g dry mass (DM).

The experimental material consisted of soluble coffee available on the Polish market (Lublin, Poland), the typical average quality coffee.

### 2.3. Willow Bark Preparation

Two g of raw material (willow bark) were poured with 15 mL of hot water; the samples were shaken for 60 min at room temperature. After centrifugation (10 min, 20°C, 4000 g), the supernatant was decanted from the precipitate, and extraction procedure was repeated. The supernatants were combined andthen the extracts were evaporated to dryness in a vacuum evaporator (50°C, under reduced pressure). Thus, obtained preparation was used for further analysis.

For extraction of water-soluble phenolic compounds 50 mg of the sample (soluble coffee,* S. myrsinifolia,* and* S. purpurea* bark preparations) were dissolved in 5 mL of warm water and were used for further analysis in order to determine the antioxidant properties of the individual raw materials. The final extracts concentration was 10 mg dry weight (DW)/mL.

### 2.4. Ultraperformance Liquid Chromatography

Compounds of interest were analyzed using a Waters ACQUITY UPLC system (Waters Corp., Milford, MA, USA), consisting of a binary pump system, sample manager, column manager, and PDA detector (also from Waters Corp.). Waters MassLynx software v.4.1 was used for acquisition and data processing. The samples were separated on a BEH C18 column (100 mm × 2.1 mm i.d., 1.7 *μ*m), which was maintained at 40°C. The flow rate was adjusted to 0.40 mL/min. The following solvent system: mobile phase A (0.1% formic acid in Milli-Q water, v/v) and mobile phase B (0.1% formic acid in MeCN, v/v) was applied. The gradient program was as follows: 0-1.0 min, 5% B; 1.0–24.0 min, 5–50% B; 24.0-25.0 min, 50–95% B; 25.0–27.0 min, 95% B; 27.0-27.1 min, 95–5% B; 27.1–30.0 min, 5% B. Samples were kept at 8°C in the sample manager. The injection volume of the sample was 2.0 *μ*L (full loop mode). Strong needle wash solution (95 : 5, methanol-water, v/v) and weak needle wash solution (5 : 95, acetonitrile-water, v/v) were used. UV-PDA data was acquired from 220 nm to 480 nm, at 5 point/s rate, 3.6 nm resolution. The separation was completed in 30 min. Peaks were assigned on the basis of their UV spectra, mass to charge ratio (*m/z*), and ESI-MS/MS fragmentation patterns.

The MS analyses were carried out on a TQD mass spectrometer (Waters Corp.) equipped with a Z-spray electrospray interface. The following instrumental parameters were used for ESI-MS analysis of phenolic compounds (negative ionization mode): capillary voltage, 2.8 kV; cone voltage, 40 V; desolvation gas, N_2_ 800 L/h; cone gas, N_2_ 100 L/h; source temperature 140°C; desolvation temperature 350°C. Compounds were analyzed in full scan mode (mass range of 100–1600 amu was scanned).

For ESI-MS/MS, selected ions were fragmented using collision energy of 15 V (phenolic acids derivatives) or 25 V (flavonoids derivatives) and collision gas (argon) at 0.1 mL/min.

### 2.5. Total Phenolic Analysis

Total phenols were estimated according to the Folin-Ciocalteu method [27]. A 0.1 mL of the extract was mixed with 0.1 mL of H_2_O, 0.4 mL of Folin reagent (1 : 5 H_2_O), and after 3 min with 2 mL of 10% Na_2_CO_3_. After 30 min, the absorbance of mixed samples was measured at a wavelength of 720 nm. The amount of total phenolics was expressed as gallic acid equivalents (GAE).

### 2.6. Determination of ABTS Radical Scavenging Activity

Free radical-scavenging activity was determined by the ABTS method according to Re et al. [28]. This reaction is based on decolourization of the green colour of the free ABTS radical cation (ABTS^•+^). The radical solution was prepared with ABTS and potassium persulfate, diluted in ethanol, at final concentration of 2.45 mM and left at dark for 16 h to allow radical development. The solution was diluted to reach absorbance measures around 0.70–0.72 at 734 nm. 1.8 mL ABTS^•+^ solution was mixed with 0.04 mL of each sample. The absorbance was measured after one minute of reaction at 734 nm. Distilled water was used as blank. Percentage inhibition of the ABTS^•+^ radical was then calculated using the equation

(1) $$\begin{array}{c} \text{scavenging}\;\%=\left[1-\left(\frac{A_{s}}{A_{c}}\right)\right]\times 100, \end{array}$$

where *A*_*s*_ is the absorbance of sample; *A*_*c*_ is the absorbance of control (ABTS solution).

All assays were performed in triplicate.

### 2.7. Inhibition of Linoleic Acid Peroxidation [29]

Ten microliters of sample was added into a test tube together with 0.37 mL of 0.05 M phosphate buffer (pH 7.0) containing 0.05% Tween 20 and 4 mM linoleic acid and then equilibrated at 37°C for 3 min. The peroxidation of linoleic acid in the above reaction mixture was initiated by adding 20 *μ*L of 0.035% hemoglobin (in water), followed by incubation at the same temperature in a shaking bath for 10 min and stopped by adding 5 mL of 0.6% HCl (in ethanol). The hydroperoxide formed was assayed according to a ferric thiocyanate method with mixing in order of 0.02 M ferrous chloride (0.1 mL) and 30% ammonium thiocyanate (0.1 mL). The absorbance at 480 nm (*A*_*s*_) was measured with a spectrophotometer for 5 min. The absorbance of blank (*A*_0_) was obtained without adding hemoglobin to the above reaction mixture; the absorbance of control (*A*_100_) was obtained with no sample addition to the above mixture. Thus, the antioxidant activity of the sample was calculated as

(2) $$\begin{array}{c} \%\;\text{inhibition}=1-\left[\frac{\left(A_{s}-A_{0}\right)}{\left(A_{100}-A_{0}\right)}\right]\times 100\%. \end{array}$$

### 2.8. Determination of Reducing Power

Reducing power was determined by the method of Oyaizu [30]. A 0.5 mL of extract was mixed with 0.5 mL (200 mM) of sodium phosphate buffer (pH 6.6) and 0.5 mL potassium ferricyanide (1% v/v) and samples were incubated for 20 min at 50°C. After that, 0.5 mL of TCA (10% v/v) was added and samples were centrifuged at 650 g for 10 min. Upper layer (1 mL) of supernatant was mixed with 1 mL of distilled water and 0.2 mL of ferric chloride (0.1% v/v). The absorbance was subsequently measured at 700 nm in the spectrophotometer.

Antioxidant activities (except reducing power) were determined as EC_50_-extract concentration (mg/mL) provided 50% of activity based on a dose-dependent mode of action. Reducing power determined as EC_50_ is the effective concentration at which the absorbance was 0.5 for reducing power and was obtained by interpolation from linear regression analysis.

### 2.9. The Isobolographic Analysis of Interactions

In order to have isobolographic analysis of interaction between the active compounds of coffee and willow bark the following samples were prepared (Table 1).

To the thus prepared mixtures 6 mL of H_2_O was added. Each type of extract was prepared in duplicate.

An isobole is an “iso-effect” curve, in which a combination of constituents (*d*_*a*_, *d*_*b*_) is represented on a graph, the axes of which are the dose-axes of the individual agents (*D*_*a*_ and *D*_*b*_). If the agents do not interact, the isobole will be a straight line. If synergy is occurring, the curve is said to be “concave.” The opposite applies for antagonism, in which the dose of the combination is greater than expected and produces a “convex” isobole.

### 2.10. Theoretical Approach

In accordance with the definition, the half-maximal inhibitory concentration (IC_50_) is a measure of the effectiveness of inhibitors. It is commonly used as a measure of antagonist drug potency in pharmacological research. The IC_50_ value is reliable for determining the activity of a single- or two-compound mixture (e.g., isobolographic analysis) [3]. Further, the EC_50_ index quantitatively measures the amount of extractor extracts mixture that is required to exhibit half of the measured activity.

The following factor was also determined [31]:

 the interaction factor (IF), which provides an explanation for the mode of interaction:
  (3) $$\begin{array}{c} \text{IF}=\frac{A_{M}}{A_{T}}, \end{array}$$

where *A*_*M*_ = measured activity of a mixture of samples and *A*_*T*_ = theoretically calculated mixture activity (based on the dose response of single components at various concentrations).

IF value <1 indicates synergistic interaction; IF > 1 indicates antagonism; IF *≈* 1 indicates additional interactions.

### 2.11. Statistical Analysis

The experimental results were mean ± S.D. of three parallel experiments (*n* = 9). Statistical analysis was performed using Statistica 7.0 software (StatSoft, Inc., Tulsa, USA) for mean comparison using Tukey's test at the significance level *α* = 0.05.

## 3. Results and Discussion

### 3.1. Identification of Coffee Phenolic Compounds

New beneficial properties of the coffee beverage are being continuously discovered [32]. Coffee brew contains many of the most important functional ingredients known, like flavonoids (catechins and anthocyanins), caffeic, and ferulic acid [33]. In addition, other biologically active compounds found in coffee are nicotinic acid, trigonelline, quinolinic acid, tannic acid, pyrogallic acid, and caffeine [34]. The beverage is also known for the antioxidant properties of its components caffeine, CGA, hydroxycinnamic acids, and melanoidins [35, 36]. Antioxidants of the hydroxycinnamic acids group, such as combined or conjugated forms of caffeic, chlorogenic, coumaric, ferulic, and sinapic acids, are also found in coffee beverage [37].

**Figure 1:**
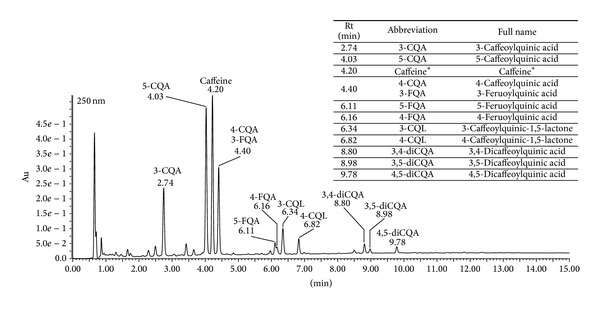
UPLC coffee extract phenolic profile.

UPLC/MS analyses allowed the identification of 11 phenolic compounds (primarily phenolic acids) in extract of used soluble coffee (Figure 1). The main phenolic acids were compounds from hydroxycinnamic acids family such as caffeoylquinic acid and its isomers. Furthermore, after detection at the wavelength at 250 nm we can observe a significant peak at retention time (Rt) 4.20 min characteristic for caffeine. Therefore, in addition to caffeine, phenolic acids are the main bioactive constituents of coffee responsible for its potential health benefits.

**Figure 2:**
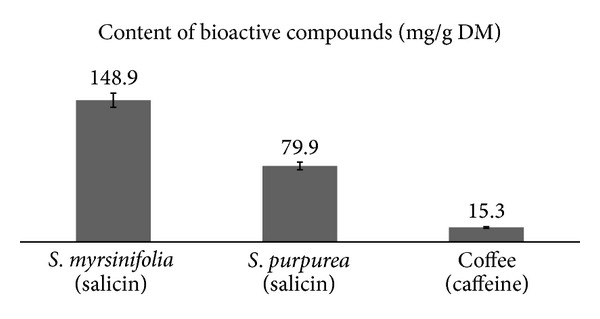
Content of distinctive bioactive compound in plant material.

The potential properties of coffee, for which the caffeine is responsible, should be mentioned. Caffeine can exert potent pharmacologic effects that can generate or alleviate headache, depending on the site of action, dosage, and timing of drug exposure. Caffeine is currently implicated in mechanisms of generation of chronic daily headache and analgesic-overuse headache. The likely target of caffeine in mediating these effects is the antagonism of adenosine receptors. Clinicians should regard caffeine as they would any other analgesic in the induction of chronic headache, and patients should be counseled to limit dietary and pharmaceutical caffeine consumption accordingly [38]. The coffee used in our experiment contained 15.27 mg/g DM of caffeine (Figure 2).

A bioactive compound is often characteristic of a plant species or even of a particular organ or tissue of the plant. This makes the dominant active compound responsible for health-promoting properties of food of plant origin. Coffee is usually associated with caffeine and its properties, and willow bark is considered a source of salicylates. In our study,* Salix *bark samples were characterized by a diverse content of phenolic glycosides (Figure 2). The purple willow (*Salix purpurea*) bark is the most important natural source of salicylic glycosides. These compounds are easily disintegrated in the gastrointestinal tract, releasing salicylic alcohol, which is in the liver oxidized to salicylic acid thereby causing no damage to the gastric mucosa [39].

### 3.2. Total Phenolics Content

According to current literature data the most condensed source of polyphenols among all beverages consumed in the world is coffee [40]. It is also confirmed by research of Svilaas et al. [41], which indicate a high coffee position among food products providing antioxidants. According to the literature 180 mL of brewed coffee provides an average of 936 mg of polyphenols [40], while other literature data indicate that one cup of coffee contains 200–500 mg of polyphenols [41]. Ramirez-Coronel et al. [42] using the HPLC method showed that one kilogram of coffe fruit pulp contains 37.9 g of polyphenolic compounds including: 11.8 g of chlorogenic acid, 20.01 g of proanthocyanidins and 0.6 g of flavonoids. The results obtained in this study indicate that the polyphenol content of the freeze-dried coffee, commercially available, is 26.71 mg/g DM (Table 2).

The reasons for the differences in the content of polyphenolic compounds in the extracts of coffee, presented in different scientific studies, are few. Researchers have used different varieties of coffee to their analysis, with different degrees of maturity and originating from different countries. The concentration of polyphenols in coffee beans depends on the species, variety, and roasting procedures [43], resulting in the decrease in the contents of polyphenols [44].

Willow's active chemical constituent, salicin, was identified in 1829 by the French pharmacist H. Leroux [45]. Salicin and salicylic acid were widely used by 19th century European physicians to treat rheumatic fever and as an antipyretic, gout remedy, and analgesic, particularly for joint pain [46]. Acetylsalicylic acid, firstly synthesized by a French chemist in 1853, was rediscovered by Felix Hoffman at the Bayer Company who created acetylsalicylic acid from the spiric acid (aspirin) found in meadowsweet in the 1890s [47]. However, the high doses used (8–10 grams daily) routinely led to vomiting and gastric irritation, and the search was on for a less noxious salicylate. Some herbalists recommend willow bark extract as a natural substitute for aspirin to achieve these same benefits. In Germany, willow bark is often taken along with aspirin to enhance the therapeutic effects while minimizing side effects [48]. The European Scientific Cooperative on Phytotherapy (ESCOP) has approved willow bark extract to treat fever, pain, and mild rheumatic complaints [49].

The healing properties of willow due to presence of salicylic glycosides, the content of which based on salicin, is strictly defined and is a minimum of 1%. Among salicylic glycosides five compounds were detected: salicin, salicortin, populin, fragilin, and tremulacin; only salicin is in the form of glycosides, and the others are in the form of glycosides, also esterified with different acyl groups [19, 50]. It is considered that the effect of anti-inflammatory and analgesic willow bark determines not only the salicylic derivatives but also other compounds present in the raw material, such as flavonoids, proanthocyanidins, and phenolic acids, which have antioxidant properties and the ability to “scavenge” free radicals [19].

Knowledge about the glycoside content in* S. myrsinifolia* is insufficient. The studies of Sugier et al. [23] show that the bark of the dark-leaved willow was characterized by the phenolic glycoside content ranging from 14.34 to 30.08% and mean value 22.38% in the year 2007, 23.67% in the year 2008, and 24.27% in 2009. The mean content of phenolic glycosides in the willow bark was higher in 2009 than in 2008 and 2007, but the differences were not statistically significant.

From among the several willow species used as herbal raw material, the bark of* S. myrsinifolia* was characterized by the highest concentration of phenolic glycosides. The content of salicylates in the bark of* S. myrsinifolia* was higher in comparison to other herbal willows, such as* S. purpurea*,* S. daphnoides*,* S. alba*, and* S. pentandra* L. [50–57]. In our study we also observed a higher level of phenolic compounds in the bark of willow* S. myrsinifolia* (23.10 mg/g DM) compared to the* S. purpurea *(20.04 mg/g DM).

Among the* Salix* spp. only* S. purpurea* and* S. alba* are recognized as medicinal plants in Poland [58]. The concentration of salicylates recorded in study of Sugier et al. [23] was many times higher than the minimum reported in Polish Pharmacopoeia VI (2002). Therefore, this species should certainly be treated as yet another taxon which can be recognized as a medicinal plant and commonly used in the pharmaceutical industry.

Most willow species constitute a basic floristic element of vegetation and are frequent in river valleys and peatlands [23, 59, 60]. Due to the high concentration of salicylates and rapid growth, many of them are recommended for herbal production and are a promising source of herbal drugs in the pharmaceutical industry [23, 54, 55, 61]. In Poland, such species as* Salix alba*,* S. daphnoides*, and* S. purpurea* originating from natural habitats and field-cultivated are mainly used to produce* Salicis cortex* [19, 54, 55].

### 3.3. Antioxidant Potential of Extracts

The antioxidant potential of plant extracts and pure compounds can be measured using numerous* in vitro* assays. Each of these assays is based on one feature of antioxidant activity, such as antiradical ability or to inhibit lipid peroxidation. However, the total antioxidant activities of food of plant origin cannot be evaluated by any single method, due to the complex nature of phytochemicals. Two or more methods should always be employed in order to evaluate the total antioxidant effects [62]. In this study ABTS decolorization assay, ferric reducing antioxidant power, and ability to lipid peroxidation inhibition were used for screening of the antioxidant activities of analyzed samples. Several phenolic acids, including salicylic and caffeic acids, possess anti-inflammatory and analgesic activity which has been associated with their antioxidant activity. The scavenging of oxygen free radicals decides about the anti-inflammatory activity of gallic and protocatechuic acids [63]. Antioxidant activity was also shown for caffeic, ferulic, and chlorogenic acids [26].

**Figure 3:**
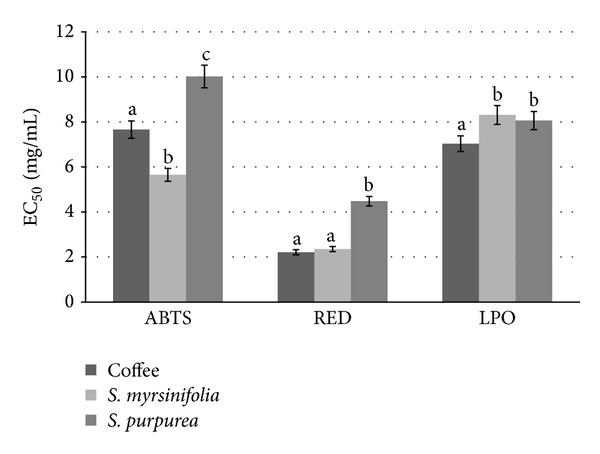
Antioxidant activities of extracts from soluble coffee,* S. myrsinifolia*, and* S. purpurea*. ABTS: antiradical activity; RED: reducing power; LPO: inhibition of lipid peroxidation. Means with different letter within a same activity are significantly different (*a* < 0.05).

Preparations of both* Salix* species bark showed significant antiradical activity (Figure 3) and the highest ability to neutralize free radicals was noted for* S. myrsinifolia* (EC_50_ = 5.65 mg/mL). Taking into account coffee extract, its antiradical activity was lower than noted for* S. myrsinifolia *but better than for extract of* S. purpurea*.* In vitro* studies on the ability of the compounds contained in the coffee extract to neutralize free radicals have shown that the effect is mainly responsible for ferulic acid, caffeic acid, and then chlorogenic acid (CGA) [10]. The studies of this group of researchers suggest that two of the most important colonic metabolites of CGA:* m*-coumaric and 3-(hydroxyphenyl) propionic acid have a high antioxidant activity. Both compounds showed antioxidant values only slightly lower than that of chlorogenic acid. Furthermore, it has been well established that CGA derivatives are the predominant antioxidants in coffee brews. Chlorogenic acids are a family of esters formed between* trans*-cinnamic acids and quinic acid. The most usual and widespread individual chlorogenic acid is formed between caffeic acid and quinic acid and the most abundant CGAs in coffee are caffeic acid including 5-caffeoylquinic acid (5-CQA) and together with two major positional isomers, 4-CQA and 3-CQA [64].

Iron salts in a biological system attach to biological molecules, where they cause site-specific formation of ^•^OH radicals and consequent damage to lipid, protein, and DNA formation of hydroxyl and peroxyl radicals (*via* the Fenton reaction) which can be delayed by chelating iron ions [65–67].

Reducing power assay measures the electron-donating capacity of an antioxidant [68]. Presence of reducers causes the conversion of the Fe^3+^/ferricyanide complex to the ferrous form which serves as a significant indicator of its antioxidant activity [69]. In general, the reducing power observed in the present study was in the following order: coffee ≥* S. myrsinifolia *>* S. purpurea*. The data presented here indicate that the marked reducing power was the highest for coffee extract and EC_50_ value was the lowest: 2.21 mg/mL. However, for* S. myrsinifolia* extract EC_50_ was very similar (2.35 mg/mL). It is presumed that the phenolic compounds may act in a similar fashion as reductones by donating electrons and reacting with free radicals to convert them to more stable products and terminating the free radical chain reaction [70].

Cell membrane phospholipids are very sensitive to oxidation and have been found to be frequent targets of radical-induced damage that enables them to participate in free radical chain reactions. Many of the fatty acids are polyunsaturated, containing a methylene group between two double bonds that makes the fatty acid more sensitive to oxidation. The high concentration of polyunsaturated fatty acids in phospholipids enables them to participate in free radical chain reactions [71]. In particular, the inhibition of lipid peroxidation (LPO) by extracts of both willows is similar (Figure 3): EC_50_ = 8.06 mg/mL for* S. purpurea* and EC_50_ = 8.31 mg/mL for* S. myrsinifolia*, respectively. Furthermore, bioactive compounds of coffee have the best ability to inhibit lipid peroxidation. In the scientific publication of Meletis [33] it has been determined that water is the best method for general antioxidant extraction. When four solvents were used, water, methanol, ethanol, and n-hexane-water extracts of coffee produced the highest yields of antioxidants and the best lipid-peroxidation protection. The water extract demonstrated a particularly high protective effect against oxidative damage to proteins. The water extract also showed superior free-radical scavenging, generally reducing the ability and capacity to bind ferrous ions thus reflecting its dynamic capacity as both a primary and secondary antioxidant. In our work, we have also analyzed activity of water-soluble bioactive compounds of coffee.

It has been proposed that although willow extracts have been traditionally used as anti-inflammatory compounds for their salicin content, the presence of high amounts of phenolic compounds can contribute to the beneficial effects seen with the consumption of commercial willow extracts [72]. We therefore propose that extracts from this species of plants may provide substantial amounts of a combination of antioxidants and thereby provide health promoting benefits to the consumers.

### 3.4. Interaction Assay

Unlike in the case of synthetic pharmaceuticals based on an activity of single (chemical) active compounds, numerous phytochemical compounds act in a beneficial manner by an additive of synergistic activity in one or numerous target sites connected to physiological processes. This idea has found an application in pharmacology during investigations on combinations of few metabolites in multidirectional therapy [73]. The method usually used for identification of interactions between active compounds is isobolographic analysis. Isobole method is independent of the mechanism of action and applies under most conditions [3].

**Figure 4:**
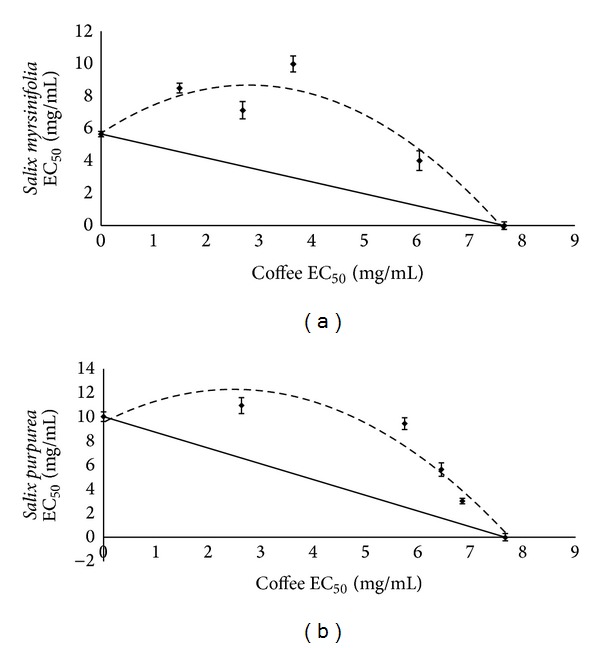
Isobole curves for 50% ABTS radical scavenging activity of coffee and willow mixtures: (a) coffee with* S. myrsinifolia*; (b) coffee with* S. purpurea*. EC_50_-extract concentration (mg/mL) provided 50% of activity based on a dose-dependent mode of action.

The antioxidant capacity of the water-soluble compounds of coffee extract in combination with willow bark extract has been revealed by ABTS method. As in Figures 4(a) and 4(b), isoboles took the convex form. This result indicates that antiradical scavengers included in coffee and willow acted as antagonists in the case of both studied species of the* Salix* family.

**Figure 5:**
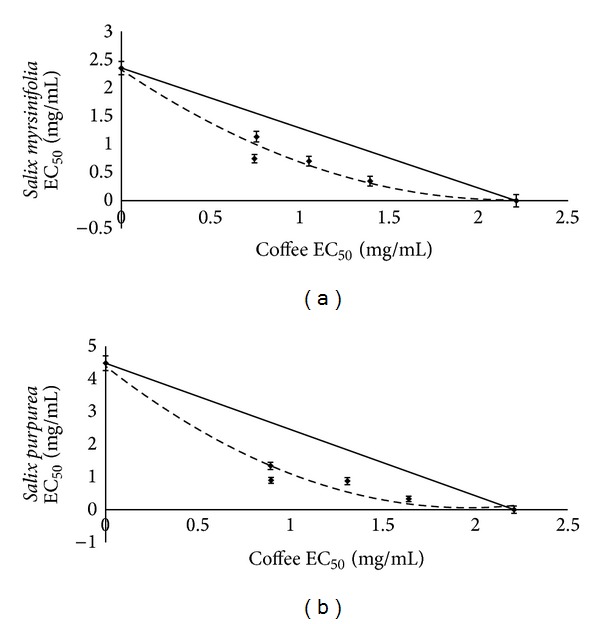
Isobole curves for 50% reducing power activity (RED) of coffee and willow mixtures: (a) coffee with* S. myrsinifolia*; (b) coffee with* S. purpurea*. EC_50_-extract concentration (mg/mL) provided 50% of activity based on a dose-dependent mode of action.

In examining the reducing power activity, we observed a synergistic interaction between coffee and both willow extracts. As Figures 5(a) and 5(b) show, isoboles have a concave shape. The results indicate that simultaneous using of drugs and/or other preparation containing studied material could give better effect than expected.

**Figure 6:**
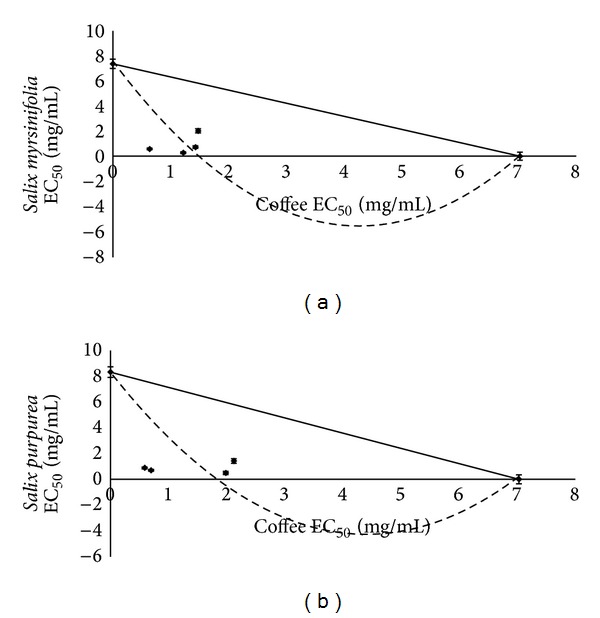
Isobole curves for 50% inhibition of lipid peroxidation (LPO) of coffee and willow mixtures: (a) coffee with* S. myrsinifolia*; (b) coffee with* S. purpurea*. EC_50_-extract concentration (mg/mL) provided 50% of activity based on a dose-dependent mode of action.

The next analysis was evaluation of ability to inhibition lipid peroxidation (LPO). As in Figures 6(a) and 6(b) isoboles took the concave form. This result indicates that bioactive compounds included in coffee and willow bark acted synergistically and effect of the combination is greater than expected from their individual dose-response curves; the dose of the combination needed to produce the same effect will be less than that for the sum of the individual components.

The isobolographic analysis is quite time-consuming and complicated; that is why we use interaction factor (IF), which provides an explanation for the mode of interaction. It is a simple way to make an assessment of type of interactions between the examined extracts or chemical compounds. As Table 3 presents, isobole curves shown in Figures 4, 5, and 6 are confirmed by the IF, calculated by the ratios of measured activity of samples and theoretically calculated mixture activity (based on the dose response of single components at various concentrations), expressed as EC_50_.

Determination of interaction factor (IF), like isobolographic analysis, is independent on mechanism of active compounds activity and requires linear relationship between an activity and sample concentration. Its crucial advantage is possibility of studies of interactions between any number of components and the fact that this is definitely less complicated. Moreover, the “strength” of interaction may be estimated approximately based on IF value. Therefore, as mentioned above, this index can be used for the rapid assessment of the interaction between the two active ingredients [31].

Phytotherapy is one of the oldest branches of conventional medicine, which is experiencing continuous growth in popularity. Using a combination of plant extracts, we can achieve better results than with one drug, often a greater dose. The key issue seems to be also minimizing or eliminating the side effects resulting from the use of medicines in smaller but therapeutically effective dose [3]. The synergism of medicinal plants is considered in three categories: synergism in the individual extracts, synergistic in herbal mixtures, and synergy in combining herbal medicine with synthetic drug. An example of synergism in herbal mixtures might be the interaction of ingredients of nettle root extract and bark of the African plum tree. The combined use of both raw extracts inhibits 5*α*-reductase and aromatase a greater extent than it would result from the sum of the activity of the individual components. In turn, in order to confirm the synergism between herbal remedy and synthetic drug, green tea with antibacterial agent ciprofloksacin was tested. Inhibition of bacterial growth and inflammation were significantly higher after administration of the drug in combination with green tea than in the case of the drug itself. This indicates a synergistic interaction of the active ingredients of tea (mainly catechins) with ciprofloksacin [74]. Williamson [3] in his work describes synergistic interactions of many herbs, including licorice (*Glycyrrhiza gabbros*), ginkgo biloba (*Ginkgo Biloba*), pepper (*Kava-Kava*), and valerian (*Valeriana officinalis*). So far in the available literature, there are no studies on the properties of coffee and willow mixtures.

## 4. Conclusion

Presented preliminary study clearly showed that both coffee and willow bark are sources of multidirectional antioxidant compounds. Additionally, phytochemicals from willow bark possessed hydrophilic character and thermostability which justifies their potential use as an ingredient in coffee beverages. It has been found that the biologically active compounds contained in the analyzed raw materials interact with each other, thus affecting their activity. Antagonism is demonstrated with respect to the ABTS radical neutralizing capacity, while with other determinations performed synergistic interaction between the active compounds, the coffee extract, and the individual willows was observed.

Proposed mixtures may be used in the prophylaxis or treatment of some civilization diseases linked with oxidative stress. Most importantly, especially strong synergism was observed for phytochemicals able to prevent lipids against oxidation, which may suggest protective effect for cell membrane phospholipids. Obtained results indicate that extracts from bark tested* Salix* genotypes as an ingredient in coffee beverages can provide health promoting benefits to the consumers; however, this issue requires further study.

## Figures and Tables

**Table 1:** Composition of samples used for isobolographic analysis.

Coffee [mg DW]	Willow bark preparation [mg DW]	Weight ratio [w/w]
100	150	2 : 3
125	125	1 : 1
150	100	3 : 2
200	50	4 : 1

**Table 2:** Total phenolic content of coffee, *S*. *myrsinifolia*, and *S*. *purpurea* samples.

Sample	Total phenolic content [mg/g DM]
Coffee	26.71 ± 1.3
*S. myrsinifolia *	23.10 ± 1.2
*S. purpurea *	20.04 ± 1.0

**Table 3:** Comparison of interaction factors (IF) of mixtures of coffee with willow bark preparation.

Sample	Willow bark	Activity	A_M_*	A_T_**	IF***
Coffee/willow bark preparation mixture (1 : 1 [w/w])	*S*. *myrsinifolia *	Antiradical potential	9.97	6.66	**1.50**
		Reducing power	1.49	2.28	**0.65**
		Inhibition of lipid peroxidation	1.27	7.55	**0.17**
	*S*. *purpurea *	Antiradical potential	5.67	8.84	**1.48**
		Reducing power	1.79	3.35	**0.54**
		Inhibition of lipid peroxidation	1.40	7.67	**0.18**

*Measured activity (*A*_*M*_) of a mixture of samples (expressed as EC_50_ [mg/mL]).

**Theoretically calculated mixture activity (*A*_*T*_) (based on the dose response of single components at various concentrations) (expressed as EC_50_ [mg/mL]).

***Interaction factor (IF) value < 1 indicates synergistic interaction; IF > 1 indicates antagonism; IF ≈ 1 indicates additional interactions.
